# Previous Antibiotic Exposure Reshapes the Population Structure of Infecting Uropathogenic Escherichia coli Strains by Selecting for Antibiotic Resistance over Urovirulence

**DOI:** 10.1128/spectrum.05242-22

**Published:** 2023-06-20

**Authors:** Gregory A. Ballash, Dubraska Diaz-Campos, Joany C. van Balen, Dixie F. Mollenkopf, Thomas E. Wittum

**Affiliations:** a Department of Veterinary Preventive Medicine, The Ohio State University, Columbus, Ohio, USA; b Department of Veterinary Clinical Sciences, The Ohio State University, Columbus, Ohio, USA; University of Texas Southwestern Medical Center

**Keywords:** antibiotic exposure, phylogroup, resistomics, uropathogenic *E. coli*, virulomics

## Abstract

Antibiotic therapy is the standard of care for urinary tract infections (UTIs) caused by uropathogenic Escherichia coli (UPEC). However, previous antibiotic therapy may impart a selective pressure that influences the population structure and pathogenic potential of infecting UPEC strains. Here, we conducted a 3-year study using whole-genome-sequencing analysis and retrospective medical record review to characterize how antibiotic exposure influenced the phenotypic antibiotic resistance, acquired resistome, virulome, and population structure of 88 UTI-causing E. coli strains from dogs. A majority of UTI-associated E. coli strains were from phylogroup B2 and clustered within sequence type 372. Previous antibiotic exposure was associated with a population shift toward UPEC from phylogroups other than the typical urovirulent phylogroup B2. The specific virulence profiles within the accessory virulome that were associated with antibiotic use were elicited by the effect of antibiotics on UPEC phylogenetic structure. Among phylogroup B2, antibiotic exposure increased the quantity of genes within the resistome and the odds of developing reduced susceptibility to at least one antibiotic. Non-B2 UPEC strains harbored a more diverse and greater resistome that conferred reduced susceptibility to multiple antibiotic classes following antibiotic exposure. Collectively, these data suggest that previous antibiotic exposure establishes an environment that provides a selective edge to non-B2 UPEC strains through their diverse and abundant antibiotic resistance genes, despite their lack of urovirulence genes. Our findings highlight the necessity for judicious use of antibiotics as we uncover another mechanism by which antibiotic exposure and resistance can influence the dynamics of bacterial infectious disease.

**IMPORTANCE** Urinary tract infections (UTIs) are one of the most common infections of dogs and humans. While antibiotic therapy is the standard of care for UTIs and other infections, antibiotic exposure may influence the pathogenic profile of subsequent infections. We used whole-genome sequencing and retrospective medical record review to characterize the effect of systemic antibiotic therapy on the resistance, virulence, and population structure of 88 UTI-causing UPEC strains isolated from dogs. Our results indicate that antibiotic exposure alters the population structure of infecting UPEC strains, providing a selective edge for non-B2 phylogroups that harbor diverse and abundant resistance gene catalogues but fewer urovirulence genes. These findings highlight how antibiotic resistance can influence pathogen infection dynamics and have clinical implications for the judicious use of antibiotics for bacterial infections.

## INTRODUCTION

Urinary tract infections (UTIs) are one of the most common bacterial infections affecting humans and companion animals (1, 2). Uropathogenic Escherichia coli (UPEC), strains of E. coli that are well-adapted to colonize the urinary tract, are the most common cause of UTIs (3). The intestinal tract is the main reservoir of UTI pathogens, where a milieu of E. coli strains from different phylogroups harbor various levels of UTI potential (4). Among these diverse strains, E. coli from phylogroup B2 dominate the UTI space, while other, non-B2 phylogroups cause a minority of infections (3, 5, 6). While previous studies suggest that specific genes account for the success of phylogroup B2, seminal work by Rasko and others found that most genes that encode virulence factors like fimbriae, pili, and secretion systems are often present among pathogenic, commensal, and laboratory strains (7). More recently, transcriptomics has revealed that genomically diverse E. coli strains from different phylogroups use the same transcriptional processes to survive in urine and initiate infection (8, 9). Collectively, these data raise the question of what contributes to the success of some UPEC strains at inciting infections and whether there are environmental drivers that alter the competitiveness of some strains.

Antibiotic therapy is the standard of treatment for UTIs in both humans and dogs (10, 11). However, antibiotic resistance is a growing issue among UPEC strains that can lead to treatment challenges, prolonging morbidity (12). Antibiotics can cause pronounced shifts in the intestinal microbiota, including strain-specific populations like E. coli (13). A common result of this dysbiotic state is the selection of antibiotic-tolerant strains that carry resistance genes (14). However, antibiotics do not cause uniform killing of all strains that lack resistance genes, as some strains survive stochastically or via other tolerance mechanisms, such as biofilm formation, low metabolic activity, or an adaptive stress response (15–17). More specifically, antibiotic exposures can influence the virulence and biofilm capacity of uropathogenic E. coli strains, which may help them evade antibiotic insult and compete for open niches (18, 19). During these states of antibiotic-induced dysbiosis, which can be quite prolonged, there are competitive dynamics between existing *E. coli* strains that maintain different antibiotic resistance and virulence potential (20, 21). Despite these known effects at the intestinal level, which is the main reservoir of UPEC, whether and how this antibiotic-induced dysbiosis influences the infecting population of UPEC strains remains an unanswered question. An understanding of how antibiotic use alters the population structure and pathogenic potential of UTI-causing UPEC strains will provide greater insight into the evolution of UTIs and how they are diagnosed and treated.

Here, we report the results of a 3-year study utilizing whole-genome sequencing, phenotypic resistance testing, and retrospective medical record review to determine whether antibiotic exposure influences the infecting strains of UPEC UTIs in dogs. Our holistic approach allowed us to determine if exposure to systemic antibiotic therapy within 30 days prior to UTIs influenced the phylogroup and pathogenomics of the infecting UPEC strains. We hypothesized that previous antibiotic exposure would reshape the population structure, skewing it toward uncommon strains that maintain robust and diverse phenotypic and genotypic antibiotic resistance but less urovirulence.

## RESULTS

### Dogs with a history of recurrent UTI are at greater odds of being exposed to antibiotics in the 30 days prior to their current UTI.

**FIG 1:**
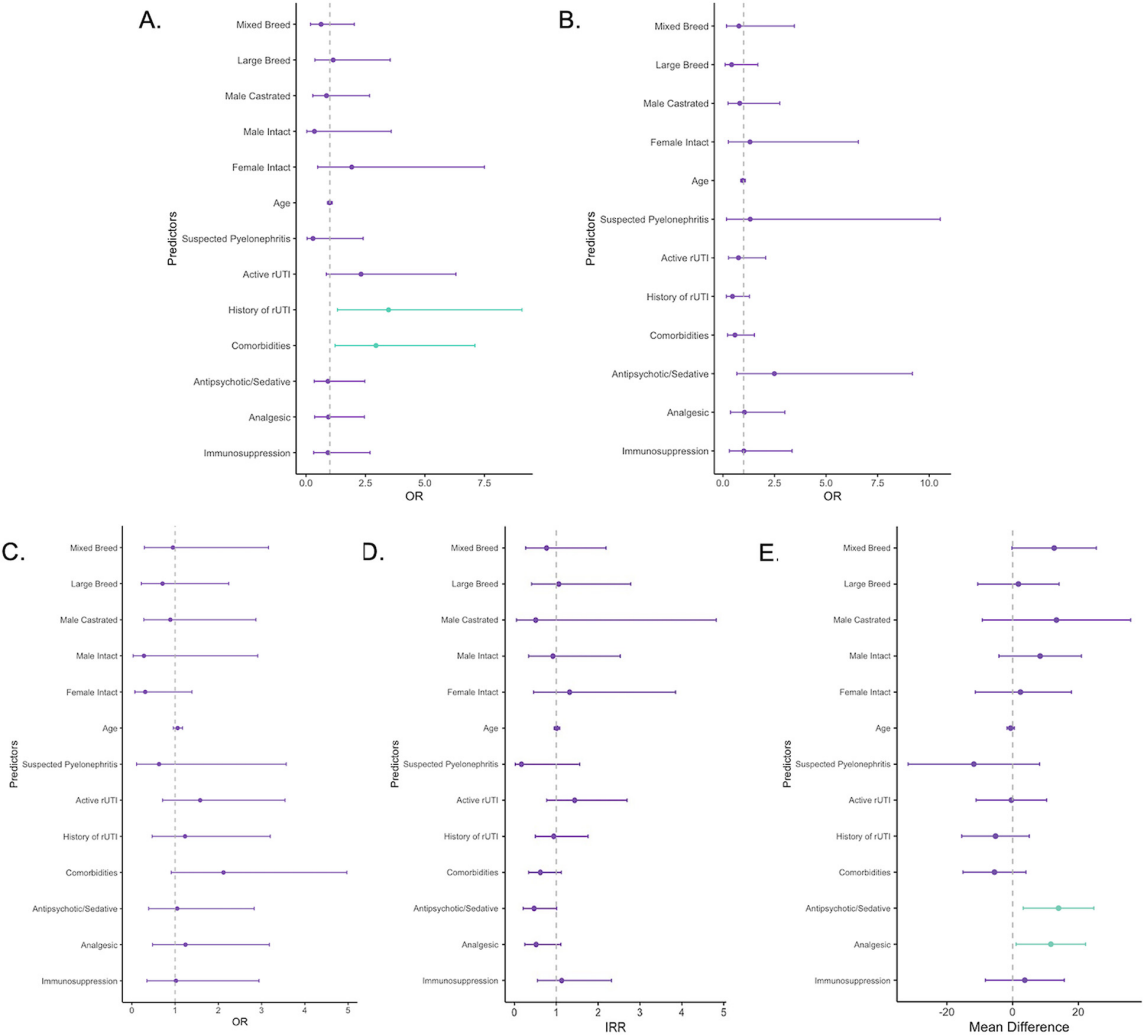
Forest plots of univariate regression models used to determine measures of association between clinical and demographic predictors and different outcome variables. (A) Comparison of the odds of clinical and demographic variables between dogs that were exposed to antibiotics in the previous 30 days and unexposed dogs. (B) Univariate analysis to determine associations between the UPEC phylogroup (non-B2 versus B2) and patient variables. (C to E) Univariate analysis to identify patient factors that were significantly associated with a reduced susceptibility phenotype (C), the number of resistance genes (D), and the number of virulence genes (E). Points and error bars represent the appropriate measure of association for each model (odds ratio [OR], incidence risk ratio [IRR]. or mean difference) and its 95% confidence interval. Gray vertical dashed lines indicate the null value for each measure of association. Values in green indicate a statistically significant association (*P* ≤ 0.05), and those in purple indicate null associations (*P* > 0.05).

Of the 124 isolates from 112 different dogs, 88 (71%) unique isolates from 79 different dogs had a complete history and medical record that allowed us to retrospectively characterize their antibiotic exposure over the previous 30 days. Eight dogs submitted repeat samples, and one dog submitted three samples over the study period. All dogs had a diagnostic sample collected by cystocentesis. Forty-three isolates (48.9%) came from patients that had received at least one antibiotic in the previous 30 days. Univariate analysis showed that the dogs exposed and unexposed to antibiotics were largely the same except for differences in the presence of comorbidities (unexposed, 16/45, 35.6%; exposed, 25/43, 58.4%; *P* = 0.017) and a history of previous UTI (unexposed, 8/45, 18.2%; exposed, 19/43, 44.2%; *P* = 0.011) (Fig. 1A). Multivariate analysis identified history of previous UTI (adjusted odds ratio [OR], 3.05; 95% confidence interval [CI], 1.11 to 8.35; *P* = 0.035) as the only significant difference between the antibiotic-exposed and -unexposed groups (comorbidities: adjusted OR, 2.24; 95% CI, 0.85 to 5.89; *P* = 0.101).

### Patient demographics and clinical factors are not associated with UPEC phylogroup, virulence, and antibiotic resistance.

A majority of the epidemiological variables collected for dogs in this study were not associated with UPEC strain phylogroup, virulence, or resistance (Fig. 1B to E). Interestingly, the virulome of UPEC strains contained more genes if dogs were currently taking an analgesic (*P* = 0.031) or antipsychotic/sedative (*P* = 0.011) compared to dogs not taking those medications. However, upon further investigation, we found that these factors confounded one another. After controlling for dogs taking analgesics at the time of diagnosis, antipsychotic or sedative use did not influence the number of virulence genes harbored by the infecting UPEC strain (adjusted mean difference, 10.68; 95% CI, −1.51 to 22.87; *P* = 0.086). Similarly, after adjusting for antipsychotic or sedative use, UPEC strains from dogs that took analgesics did not carry significantly more virulence genes (adjusted mean difference, 6.59; 95% CI, −5.28 to 18.47; *P* = 0.276). Since patient demographics and clinical factors did not alter the phylogenetics and pathogenomics of the UPEC strains, these variables were excluded from all subsequent multivariable models investigating phylogroup, virulome, resistome, and reduced-antibiotic-susceptibility outcomes.

### Antibiotic use modulates the phylogroup structure of UTI-associated E. coli strains.

**FIG 2:**
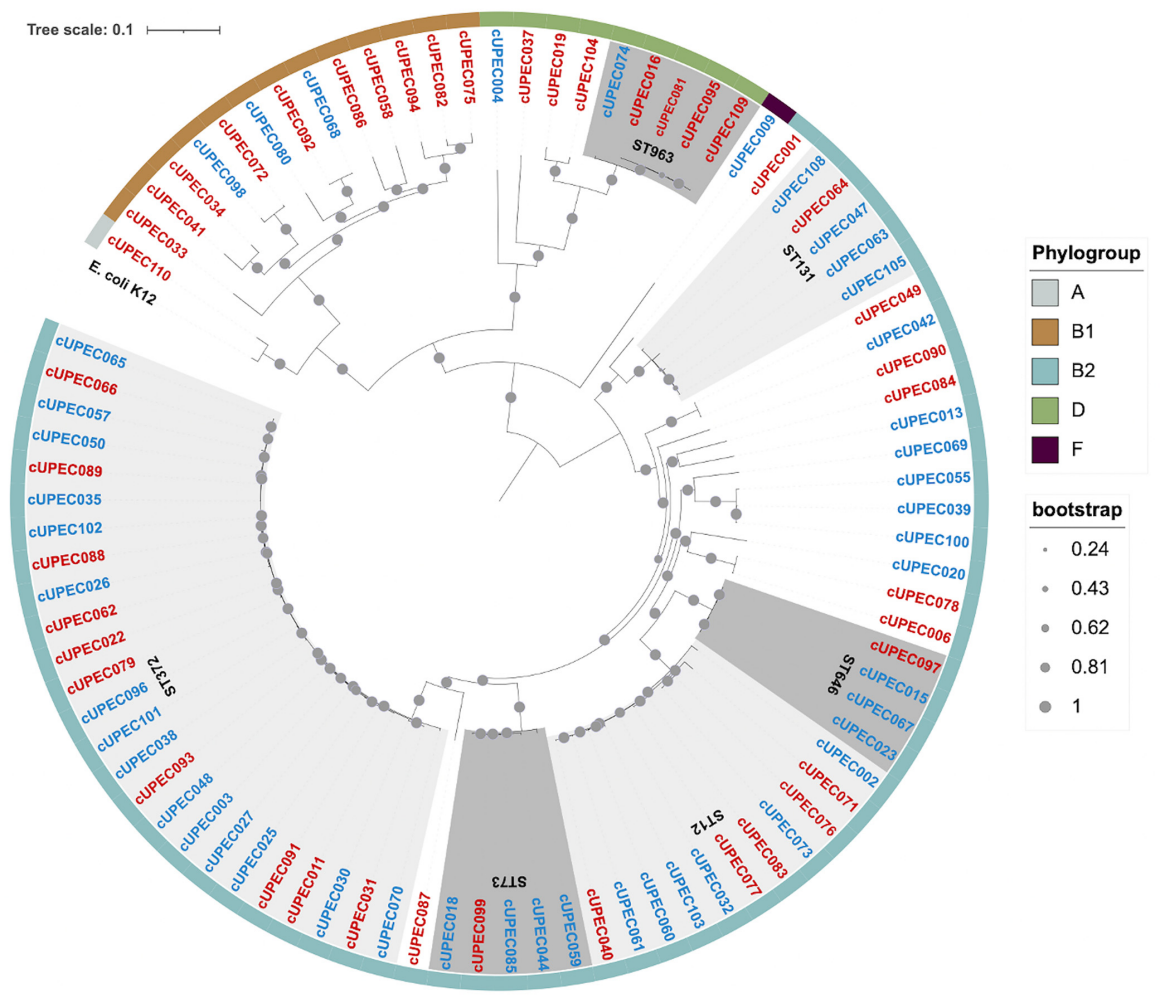
*De novo* core-genome SNP phylogenetic analysis of 88 canine UPEC (cUPEC) isolates collected over a 3-year period. Isolates clustered by phylogroup (outer ring) and Achtman sequence type/sequence type clusters (gray shading). Isolates that were exposed to systemic antibiotics within the previous 30 days are colored red, and those not exposed within the previous 30 days are blue.

Core-genome single-nucleotide polymorphism (SNP) phylogeny revealed a UPEC population structure among the dogs that was segregated into six phylogroups, with 1.1% (1/88) for phylogroup A, 14.8% (13/88) for B1, 72.7% (64/88) for phylogroup B2, 10.2% (9/88) for phylogroup D, and 1.1% (1/88) for phylogroup F (Fig. 2). Further classification identified 36 different sequence types (STs). Despite this diversity, more than 60% of the sample population clustered within 6 sequence types: sequence type 372 (ST372) (24/88, 27.3%), ST12 (11/88, 12.5%), ST963 (5/88, 5.7%), ST73 (5/88, 5.7%), ST131 (5/88, 5.7%), and ST646 (4/88, 4.5%).

Among dogs receiving antibiotic therapy in the previous 30 days, 25.6% (11/43) of UPEC strains were in phylogroup B1, 58.1% (25/43) were in B2, and 16.3% (7/43) were in D (Fig. 2). Dogs that received antibiotics within the previous 30 days were at 4.68 times the odds (95% CI, 1.63 to 13.39; *P* = 0.004) of having an E. coli strain from a phylogroup other than B2 compared to dogs that did not receive antibiotics. Population structure was not influenced by sex, breed, age, comorbidity status, the use of analgesics, immunosuppressants, or sedatives/antipsychotics, or the presence or history of recurrent UTIs.

### Virulomics is driven by phylogroup association independent of antibiotic use.

**FIG 3:**
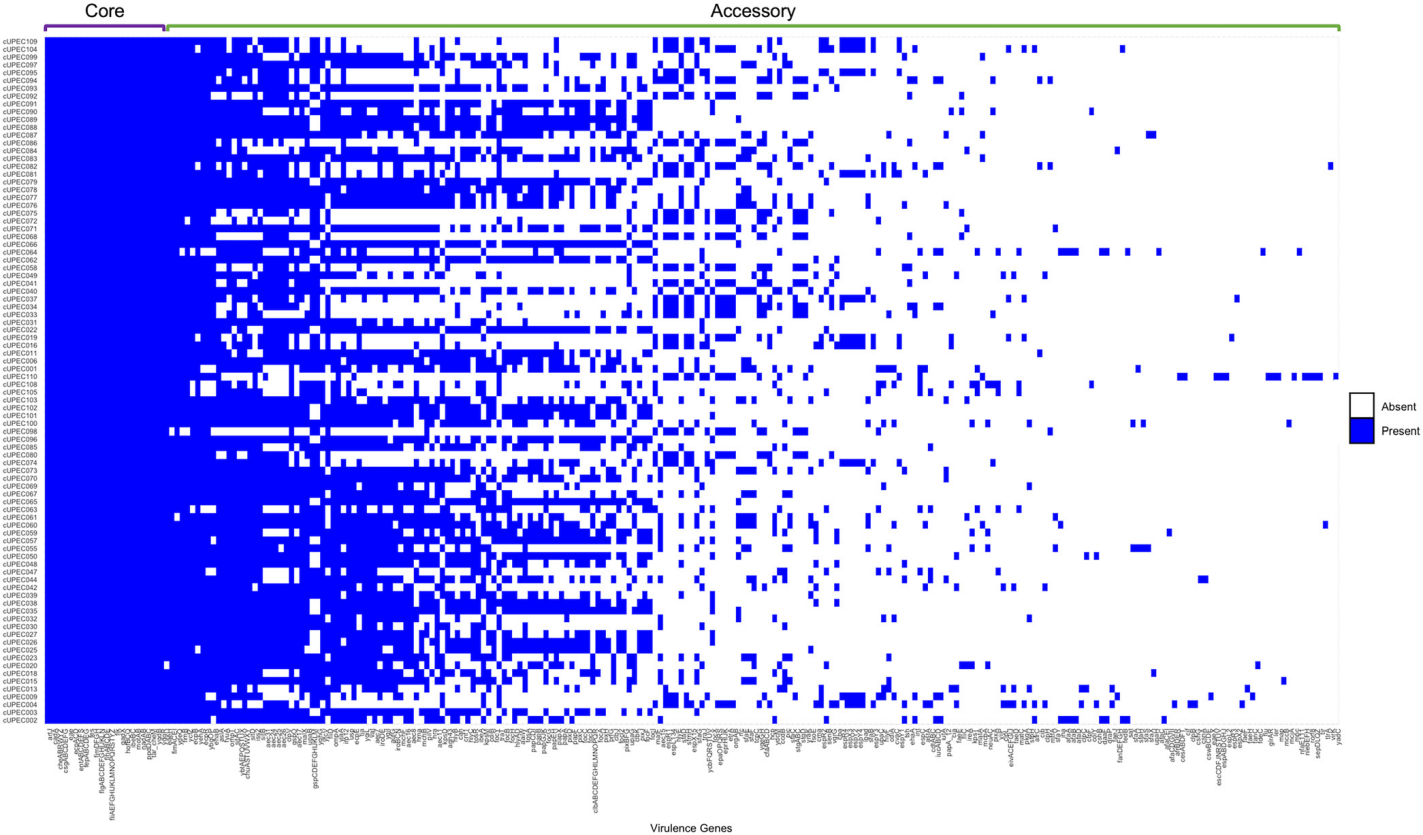
The individual virulomes of all 88 cUPEC isolates. The core virulome in this sample consists of 91 genes (20% of the virulome), including genes associated with iron acquisition, amino acid metabolism and transport, motility, and fimbria/pilus assembly. The accessory virulome was composed of 365 genes (80%) and was quite variable among the population. The accessory genome consisted of genes necessary for colonization and invasion of the urothelium, including those necessary for fimbria/pilus assembly, toxin production, and iron uptake. Genes with similar proportions are combined to reduce the size of the *x* axis and are not reflective of functional operons.

Each UPEC isolate was screened for 456 virulence genes, including fitness factors, known to contribute to E. coli pathogenesis. The core virulome consisted of 91 (20.0%) virulence genes, most of which contributed to iron acquisition (*ent* and *fep*), amino acid metabolism and transport (*artJ* and *cadA*), motility (*fli*, *flg*, and *flh*), and fimbrial assembly *(ecp*, *csg*, and *fim*) (Fig. 3). The accessory genome consisted of 365 genes of differing prevalence among the UPEC isolates. The number of virulence genes ranged from 156 to 248, with an average of 208 ± 23 genes (mean ± standard deviation).

**FIG 4:**
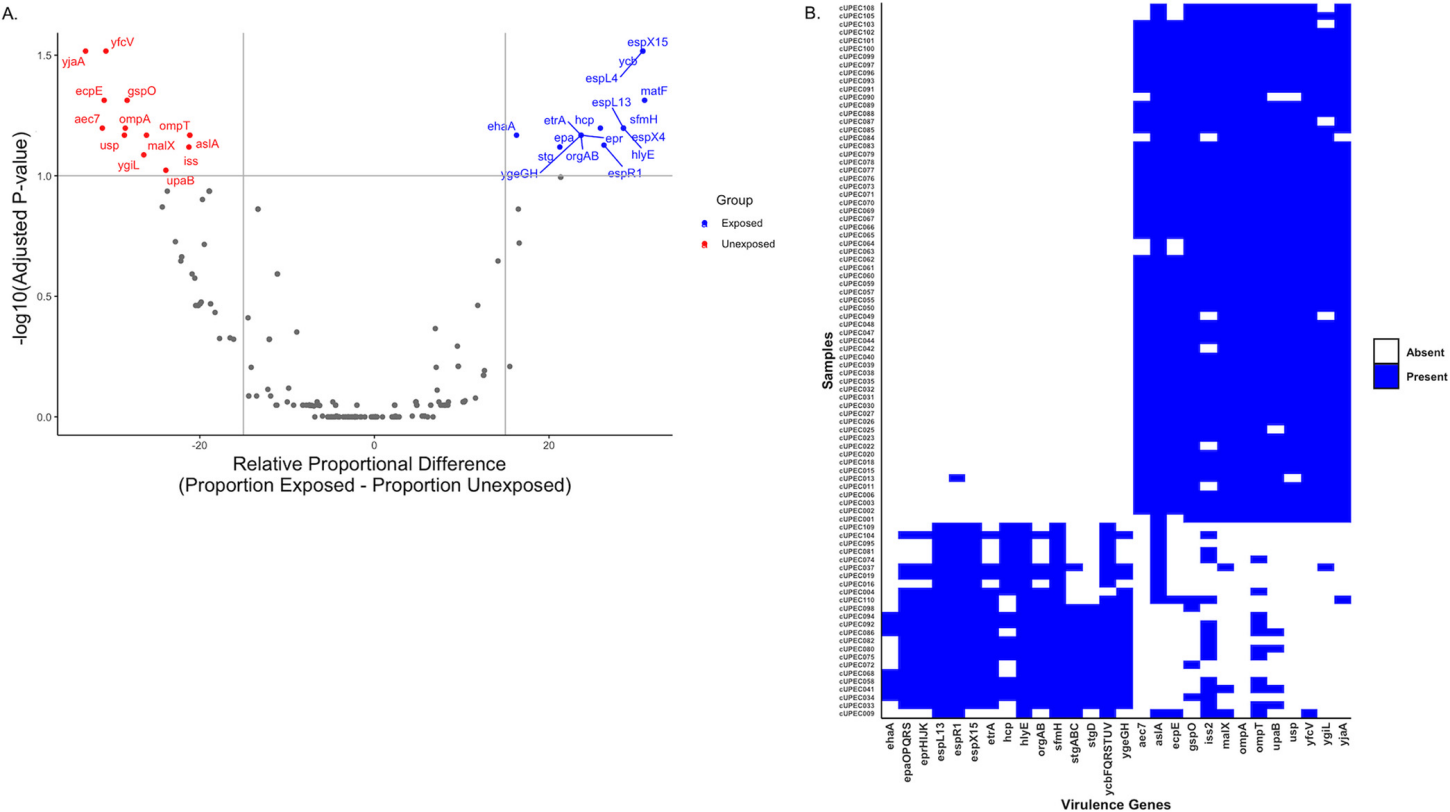
Differences in accessory virulence genes across antibiotic exposure status and phylogeny. (A) Volcano plot showing virulence genes at higher relative proportions among dogs that were not exposed to antibiotics in the previous 30 days (red) and those that were (blue). (B) The virulence genes that were differentially abundant across antimicrobial status were often dictated by phylogroup (non-B2 versus B2). Genes with similar proportions are combined to reduce the size of the *x* axis and are not reflective of functional operons. Only genes that had a relative proportional difference of 15%, an unadjusted *P* value of <0.05 by Fisher’s exact test, and an adjusted *P* value of <0.01 by the Benjamini-Hochberg correction are shown.

A total of 37 genes were significantly more prevalent among UPEC strains from dogs that were exposed to antibiotics, compared to 21 genes that were more abundant among UPEC strains from dogs unexposed to antibiotics (adjusted *P* value of <0.1) (Fig. 4A). Abundant genes in the unexposed group included fimbrial (*yfcV* and *ygiL*), autotransporter (*upaB* and *usp*), porin (*ompA* and *ompT*), and fitness (*aslA*, *iss2*, and *malX*) genes associated with extraintestinal infections and UTIs. Those upregulated in the unexposed group included genes involved in secretion systems (*espL*, *espR*, *espX*, *epa*, *epr*, *org*, and *hcp*) and fimbriae (*ycb*, *stg*, *yge*, and *sfmH*) and other genes (*hlyE* and *ehaA*) that are associated with intestinal colonization and pathogenicity. Because the B2 phylogroup is associated with UTIs and other extraintestinal infections and other phylogroups are associated with intestinal colonization, we tested to see if there was a phylogroup effect among the 58 differentially abundant genes. Among those genes, 56 were significantly associated with either the B2 or the non-B2 phylogroups (Fig. 4B).

**FIG 5:**
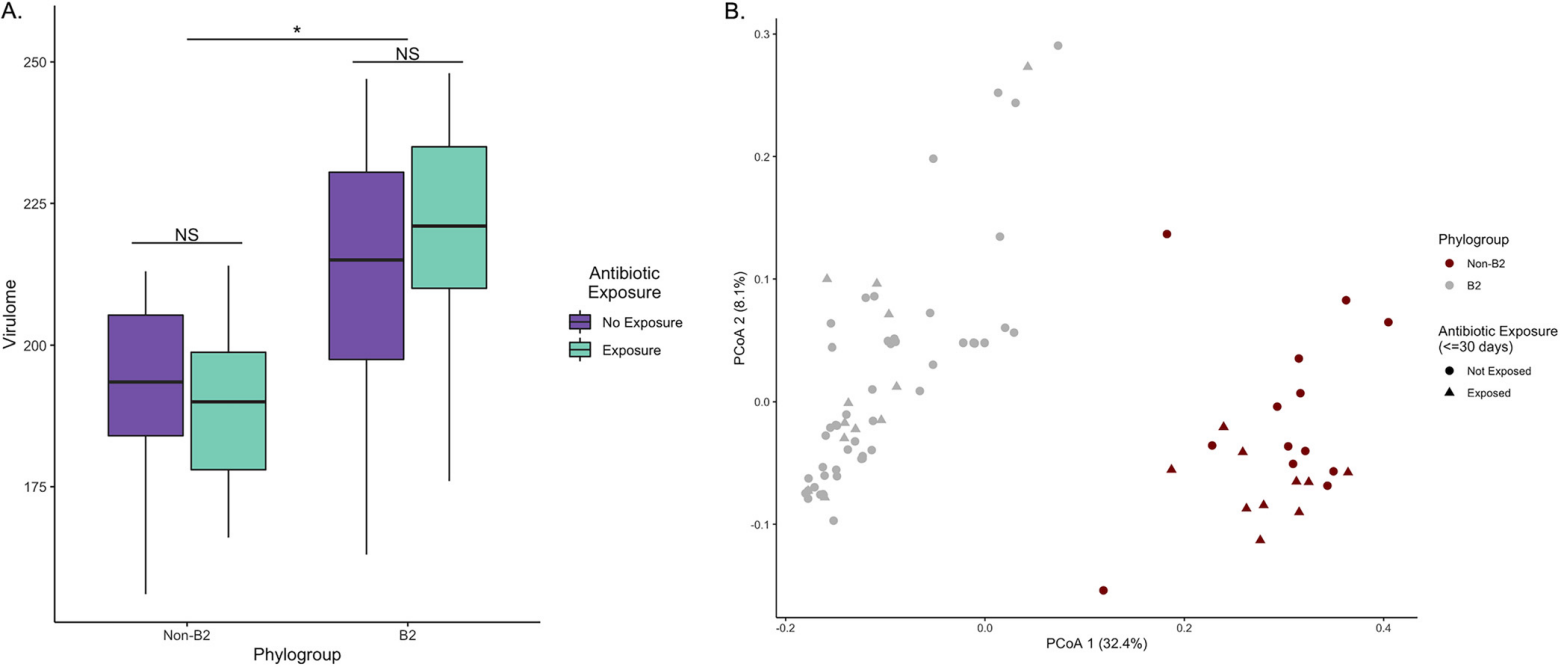
The virulome was dependent on phylogroup and independent of antibiotic exposure within the previous 30 days. (A) UPEC strains from phylogroup B2 were more likely to harbor virulence genes, on average about 25 more. Virulence gene quantity was not influenced by antimicrobial exposure. (B) Principal-coordinate analysis (PCoA) of Bray dissimilarity distances of the virulome further identified phylogroup as a discriminating factor for virulence clustering. Antimicrobial exposure did not identify distinct clusters after plotting PCoA. NS, not significant; *, *P* < 0.05.

We then assessed whether the overall virulomic profile of canine UPEC was driven by the isolates’ phylogroup independent of antibiotic use. On average, infecting UPEC strains from phylogroup B2 carried approximately 27 more genes (β, 27.2; 95% CI, 18.06 to 36.26; *P* < 0.001) than non-B2 UPEC strains after controlling for antibiotic exposure. Contrastingly, antibiotic exposure within the previous 30 days did not significantly alter virulence gene carriage after controlling for phylogroup status (β, 5.40; 95% CI, −3.55 to 14.34; *P* = 0.233) (Fig. 5A). Using the virulome to construct principal-coordinate analysis (PCoA) of Bray-Curtis dissimilarity distances, we identified distinct virulomic clustering that was dependent on phylogroup association and independent of antibiotic exposure (Fig. 5B).

### Antibiotic exposure is associated with the acquired resistome in a phylogroup-dependent manner.

**FIG 6:**
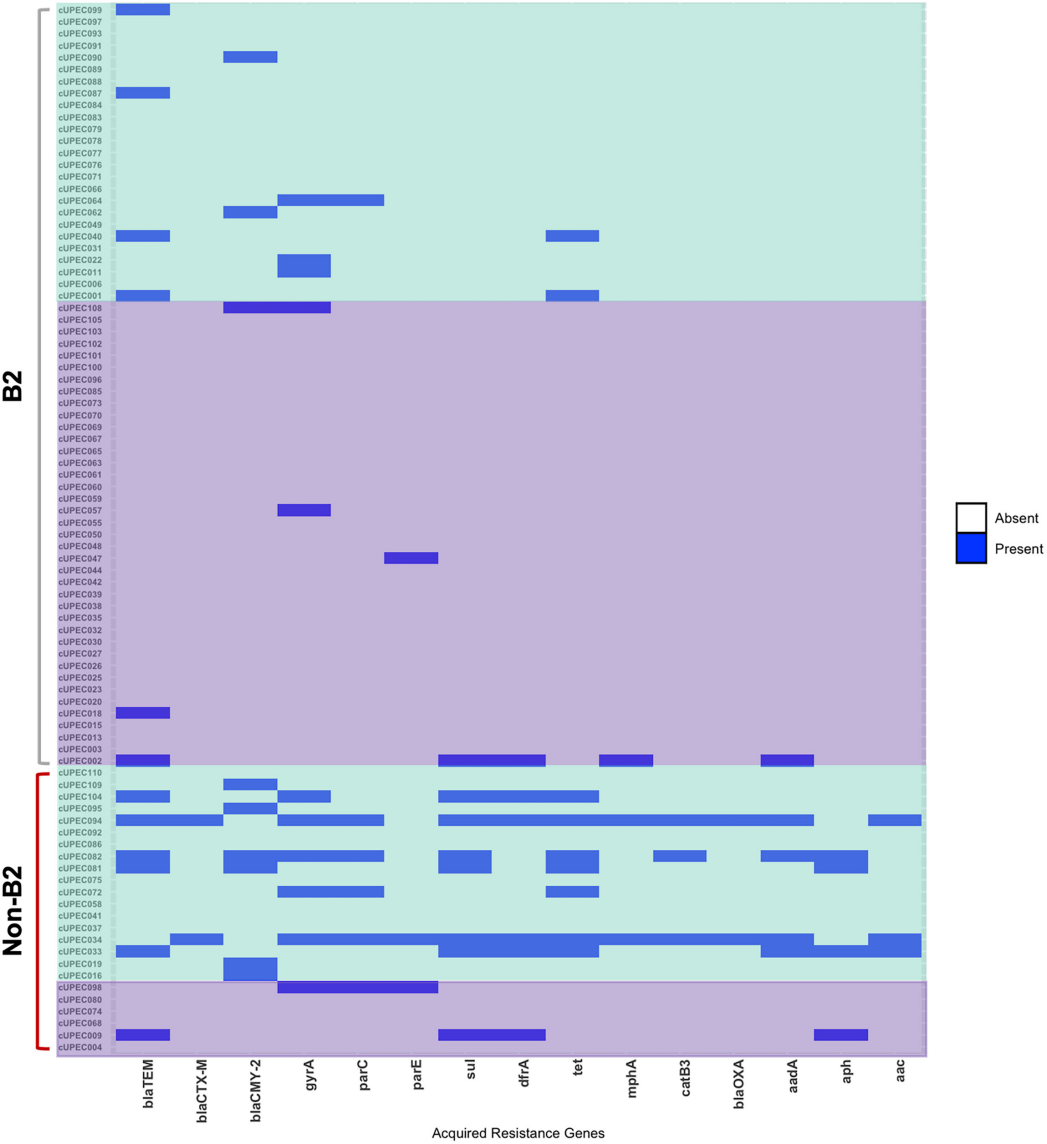
The acquired resistomes of the 88 UPEC strains in this study. Samples are clustered by phylogroup (non-B2 and B2) and then by antibiotic exposure (purple, not exposed; green, exposed).

The quantity of acquired resistance genes among UPEC strains was significantly greater among dogs that were treated with antibiotics within 30 days of their UTI (*z* = −3.1; *P* = 0.0019) (Fig. 6). UPEC isolates from phylogroups other than B2 carried more acquired resistance genes in their resistomes than isolates from phylogroup B2 (*z* = 3.5; *P* = 0.0005). Both antibiotic exposure (OR, 3.62; 95% CI, 1.26 to 10.40; *P* = 0.017) and being in phylogroups other than B2 (OR, 2.96; 95% CI, 1.03 to 8.52; *P* = 0.044) increased the odds of harboring resistance genes. The resistome was greatest among phylogroup B1, with an average of 3.9 resistance genes per isolate and a range of 0 to 14.

The effect of previous antibiotic use on the odds of harboring a resistance gene and the number of resistance genes in the resistome were different between phylogroups. Those UPEC strains in phylogroup B2 that were exposed to antibiotics were at greater odds of carrying a resistance gene (*P* = 0.036) and carried more resistance genes (*P* = 0.0359) than B2 UPEC strains not exposed to antibiotics. Contrastingly, there was no effect of antibiotic exposure on the likelihood of carrying resistance genes or the quantity of resistance genes among UPEC strains in phylogroups other than B2 (*P* > 0.05).

### Reduced antibiotic susceptibility is associated with previous antibiotic use and phylogroup.

**FIG 7:**
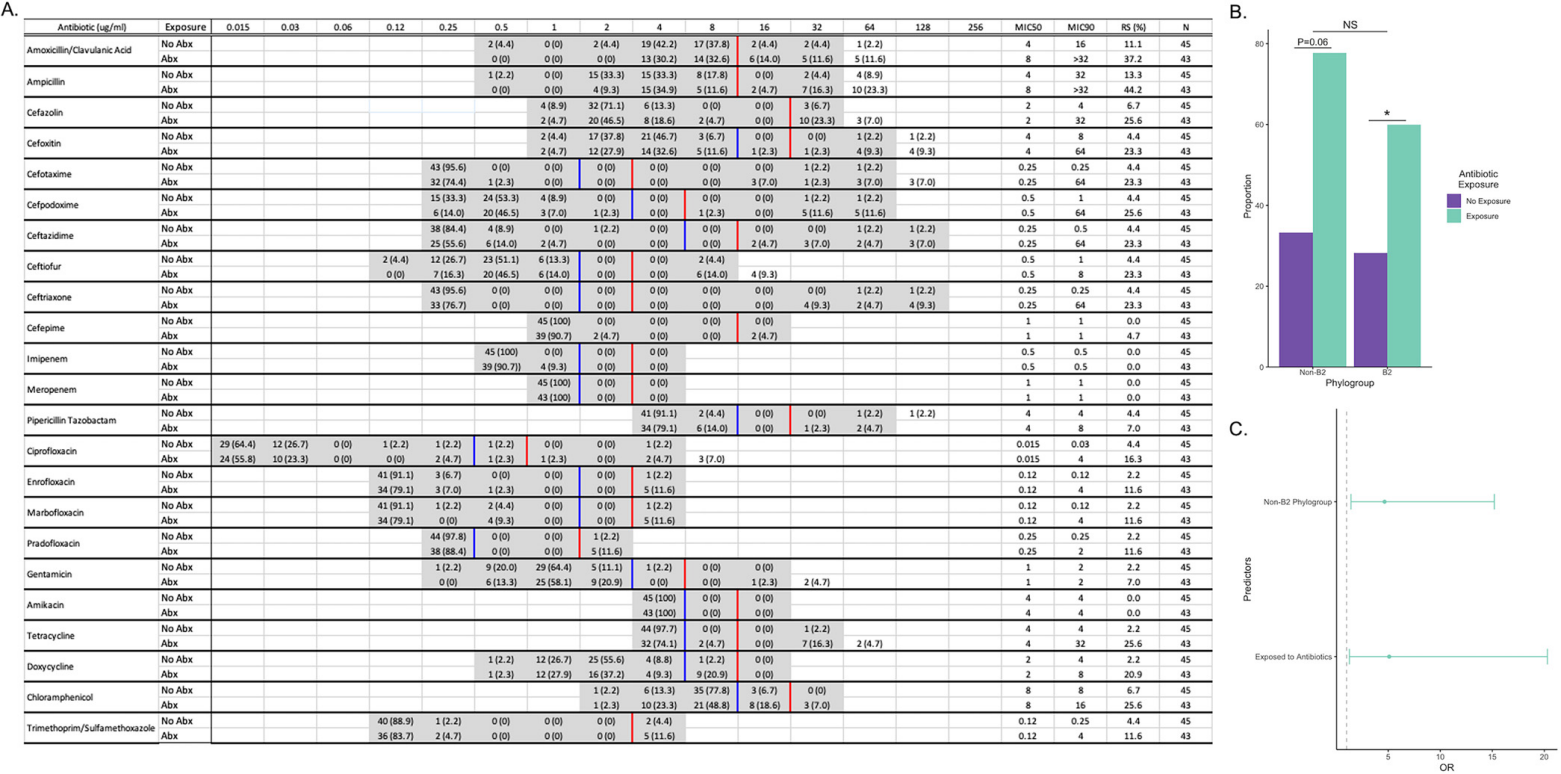
Antimicrobial exposure is associated with reduced susceptibility in a phylogenetics-dependent manner. (A) Antibiogram of UPEC isolates to 23 antibiotics of clinical importance stratified by antibiotic exposure (Abx). In most cases, there was a shift toward a higher MIC to inhibit 90% of the UPEC strains tested and a greater proportion of UPEC strains with reduced susceptibility if the patient was previously exposed to antibiotics. The tested dilution range for each antibiotic is shaded in gray. Blue and red lines indicate “Intermediate” and “Resistant” breakpoints, respectively. Reduced susceptibility is defined as those isolates with “Intermediate” or “Resistant” classification. (B) The association of antimicrobial exposure with reduced susceptibility was more pronounced in phylogroup B2. (C) Multidrug reduced susceptibility is associated with previous antibiotic exposure and being in a phylogroup other than B2. NS, not significant; *, *P* < 0.05.

Each isolate was tested against a suite of 23 antibiotics of clinical relevance to characterize phenotypic reduced susceptibility. Forty-two isolates (47.7%) had reduced susceptibility to at least one drug tested. The rates of reduced susceptibility were highest for ampicillin (25/88, 28.4%) and amoxicillin-clavulanic acid (17/88, 23.9%) (Fig. 7A).

The odds of having an infection with reduced susceptibility was 4.4 times higher among dogs that had received antibiotic therapy within the previous 30 days compared to those that did not have treatment (95% CI, 1.71 to 11.19; *P* = 0.002). While the phylogroup classification was not associated with reduced susceptibility among the strains tested (*P* = 0.249), the effect of antibiotic exposure varied within the phylogroup groupings. Antibiotic exposure was significantly associated with reduced susceptibility in phylogroup B2 (*P* = 0.013), while it was only marginally associated with reduced susceptibility in other phylogroups (*P* = 0.06) (Fig. 7B).

Eighteen (20.7%) of the isolates were resistant to drugs from three or more categories. Similar to reduced susceptibility, multidrug reduced susceptibility (MRS) was associated with previous antibiotic exposure (OR, 5.17; 95% CI, 1.29 to 20.65; *P* = 0.020) (Fig. 7C). However, in this model, isolates from phylogroups other than B2 were at 4.68 times the odds of being MRS compared to strains from phylogroup B2 (95% CI, 1.43 to 15.30; *P* = −0.011). In addition, the association between antibiotic exposure and MRS was more pronounced in UPEC strains from phylogroups other than B2 (*P* = 0.016).

### Recurrent UTIs are caused by diverse UPEC strains with differing virulomes and resistomes.

**FIG 8:**
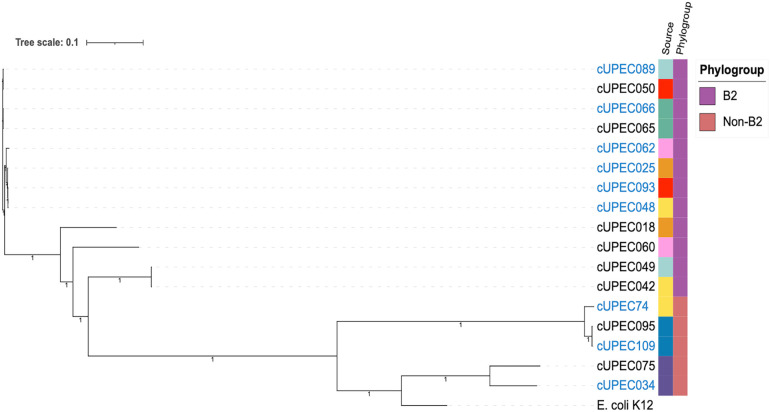
Maximum-likelihood phylogenetic tree constructed using core whole-genome SNP analysis of UPEC strains from repeat submissions by the same animals over the course of the study period. Repeat UTI submissions are identified by blue font. UPEC strains from the same patient are identified by same-color tiles in the first color strip (Source). Bootstrap values are presented on the respective branches.

Among the nine dogs that submitted more than one isolate during the study, we found that most dogs were reinfected by a new UPEC strain (7/9), but two dogs were reinfected with a clonal strain (Fig. 8). It was rare for dogs to have repeat infections with UPEC strains from different phylogroups, but one dog was infected with a B1 strain after initial B2 infection.

Among our subset of dogs with repeat isolates, we observed trends similar to those noted in our larger sample: antibiotic exposure within the previous 30 days was associated with a propensity away from phylogroup B2, increased resistance, and decreased virulence. UPEC strains isolated from repeat infections did not differ in their quantities of virulence or resistance genes or their antimicrobial susceptibilities. We did observe an inverse trend in the virulomes and resistomes among nonclonal repeat isolates, but this trend was not statistically significant.

## DISCUSSION

We report one of the first large-scale, whole-genome surveys of uropathogenic E. coli isolates from animals and explore how antibiotic exposure influences the population structure and pathogenic profiles of UTI-causing strains. Our data support our hypothesis that exposure to systemic antibiotics can alter the population structure of infecting UPEC strains away from the typical urovirulent phylogroup B2 toward other, less-well-adapted phylogroups. This observation is circumstantially supported by others who found that dogs with UTI-associated E. coli strains with common urovirulence genes were less likely to be previously exposed to antibiotics (3). It is well established that antibiotic use can elicit an ecological disturbance in the gut microbiome that alters community structure, E. coli strain dynamics, and resident E. coli strain turnover (22–24). Our findings indicate that these effects can last for a considerable duration following treatment and may influence infectious-strain dynamics in extraintestinal niches, especially those in which the intestine serves as a major reservoir of infecting strains.

Antibiotic exposure was associated with increased harborage of antibiotic resistance genes and reduced susceptibility, including reduced susceptibility to multiple drug classes. Phylogroup was also associated with antibiotic resistance, where strains belonging to non-B2 phylogroups had more robust resistomes and were at greater odds of having reduced susceptibility to multiple drugs compared to B2 UPEC strains. In the canine gut and urinary tract, antibiotic resistance is largely restricted to non-B2 phylogroups, a finding similar to previous observations in humans that commensal phylogroups are more likely to harbor antibiotic resistance (25, 26). One of the most common empirical therapies for UTIs in canine medicine is amoxicillin with or without clavulanic acid (11). The reduced susceptibility pattern and resistome largely reflected this use, as many isolates harbored one or more *bla* genes that conferred resistance to ampicillin and amoxicillin-clavulanic acid. Surprisingly, the proportion of UPEC strains with reduced susceptibility to amoxicillin was similar to recent estimates for UTI recurrence in dogs and that which is seen in this study (~20 to 30%) (27). Our data also suggest that other first-line antibiotics, such as trimethoprim-sulfonamides, may be more effective for empirical therapy, as resistance is relatively low. More research is needed to understand how antibiotics and their prescribing habits influence UPEC pathogenesis and clinical progression.

The UPEC community virulome was quite diverse, as only 20% of the 456 genes were present among all strains. Collectively, our data indicate that virulence gene prevalence was dictated by phylogroup association. Not surprisingly, B2 UPEC strains were enriched with typical urovirulence genes necessary for colonization of the bladder, while non-B2 UPEC strains harbored genes associated with colonization of the intestines. The types of virulence factors that cluster by phylogroup also correlate well with the evolutionary emergence of these strains, as a majority of the virulence factors required to colonize extraintestinal niches clustered within phylogroup B2, while those necessary for colonization of the intestine as pathogens or commensals clustered in later derived phylogroups (28, 29). At the phenotypic and molecular levels, antibiotic use can augment UPEC pathogenicity through the transfer of pathogenicity islands between bacteria via mobile genetic elements (30, 31). However, here we show that the use of antibiotics is driving alterations in virulence by selecting for specific population shifts toward less urovirulent phylogroups.

None of the patient’s clinical characteristics were associated with population structure, virulome or resistome quantity, or phenotypic resistance. We expected that dogs with comorbidities or those taking immunosuppressants would be more likely to be colonized by less virulent commensal strains. Humans with chronic conditions developed bloodborne infections with strains of E. coli that were less virulent (32). Moreover, dogs on immunosuppressants were less likely to be colonized by isolates with a UPEC pathotype (3). As artificial intelligence and risk models gain more popularity as predictive diagnostic tools, future studies should focus on standardized, repeatable methods of assessing factors that contribute to first-time and recurrent UTIs.

The uropathogenic E. coli strains collected in our study were largely classified as ST372 within phylogroup B2. Our study reiterates previous findings of phylogroup distribution among canine UPEC strains and provides rigorous evidence that ST372 is a canine-adapted UPEC sequence type (33, 34). Other common STs included ST12, ST73, and ST131, all three of which are among the top 15 globally disseminated extraintestinal sequence types known to cause human disease (35). ST12 and ST73 were previously described as human-adapted strains; however, our data, along with those of LeCuyer et al. and Valat et al., suggest that ST73 and ST12 are common sources of UTI-causing strains (25, 33). The host flexibility of UPEC sequence types echoes the concern about zoonotic transfer of UPEC strains between humans and dogs (36). The *bla*_CTX-M_-harboring ST131 E. coli is a pandemic UPEC strain that is responsible for the emergence of multidrug-resistant UTIs among humans (37). Our sequence data suggest that this pandemic strain is not well established in the study population, because despite isolating ST131, the CTX-M enzyme was only found in ST162 and ST8492 strains.

Our study has limitations that should be considered when interpreting our results. The canine population used in this study was from a tertiary referral veterinary clinic that may not be reflective of the general canine population. To account for this, we tried to utilize clinical cases that came from the hospital’s community practice service, which sees patients for preventive and outpatient services similar to what would be practiced in private settings. Our study also investigates genotypes based on draft genome sequences that could miss specific virulence or resistance genes during the assembly process. By implementing quality control cutoffs during the sequencing and assembly process, we took steps to reduce the frequency and likelihood of these errors. Future studies that pair urinary and fecal samples pre- and post-antibiotic exposure will also shed light on the mechanisms that support bladder colonization with atypical UPEC strains. Additionally, studies investigating the transcriptional and/or proteomic profiles of UPEC strains from UTIs pre- and post-antibiotic exposure are needed. It is known that diverse UPEC genotypes take a uniform transcriptional approach during UTI, but the influence of antibiotic exposure on this transcriptional profile has not been explored (8).

Collectively, our data support the hypothesis that antibiotic use creates a dysbiotic disturbance that reshapes the E. coli population toward antibiotic-resistant strains. Reports suggest that antibiotics can cause intestinal microbiome disturbance for at least 30 days, during which time competitive warfare occurs among bacteria and other microorganisms (38, 39). During this time, we believe non-B2 phylogroup strains obtain a competitive advantage via their diverse lexicon of antibiotic resistance genes that allows them to bloom in the intestinal microbiome. In addition, the virulence genes present in these non-B2 phylogroup strains also promote their success in the intestine, allowing them a further competitive advantage to colonize intestinal niches and become the dominant E. coli strains. Through sheer numbers, these strains are able to gain traction in the lower urinary tract over the more virulent and fit B2 phylogroup strains (4). However, our data suggest that B2 strains acquire resistance genes in the face of antibiotic exposure, and in doing so, may regain their competitive advantage by blooming alongside non-B2 strains, in addition to outcompeting them in the urinary space by maintaining virulence and fitness genes that contribute to their survival and urothelial invasion. While the phenomenon of antibiotic use selecting for tolerant strains is not novel, here we show that this selection process can alter the strain dynamics and disease mechanisms underlying UPEC UTIs. Moreover, these events can take place for at least 30 days following the withdrawal of antibiotic therapy. Understanding how ecological, host, and pathogen attributes contribute to intestinal colonization and infection dynamics in the bladder has implications for the management of recurrent UTIs and the development of novel, antibiotic-sparing UTI treatments that target common fitness and virulence genes.

## MATERIALS AND METHODS

### Sample collection.

Canine E. coli isolates were collected over a 3-year period from diagnostic cultures of patients with a confirmed clinical UTI in which E. coli was considered the causative pathogen. Isolates were selected to represent diagnostic E. coli isolates recovered from UTIs by the OSU Veterinary Medical Center Diagnostic and Clinical Microbiology Laboratory. All isolates were confirmed to be E. coli at the time of diagnosis and prior to molecular and phenotypic characterization using matrix-assisted laser desorption ionization–time of flight mass spectrometry (MALDI-TOF MS). A total of 124 unique isolates from 112 different dogs were collected for this study. Isolates were frozen in a 1:1 mixture of tryptic soy broth and 60% glycerol at −80° C until phenotypic and molecular characterization. This project was determined to be exempt from IACUC review because the samples were post-diagnostic specimens originally collected for another purpose.

### Retrospective medical record review and patient clinical information.

The medical record for each of the 112 different dogs included in the study was reviewed for patient information including age, breed, sex/neutering status, comorbidities known to increase the risk of UTI, history of a recurrent UTI, current recurrent UTI state, which was defined using guidelines previously established in veterinary medicine, current use of antipsychotics/sedatives, analgesics, and/or immunosuppressive medication, diagnosis or suspicion of pyelonephritis, and antibiotic use within the previous 30 days (9). Comorbidities, history of recurrent UTI, current recurrent UTI state, use of antipsychotics/sedatives, analgesics, or immunosuppressive medication, pyelonephritis, presence of clinical signs, and previous antibiotic use were collected as binary data points. Sex/neutering status was categorized as intact female, spayed female, intact male, and castrated male and breed as pure small breed (<12.5 kg), pure large breed (>12.5 kg), and mixed breed. Age was collected as a continuous variable. Dogs that lacked a complete history or medical record or that could not be traced back 30 days from their initial infection using their medical record were removed from consideration in the study. Diagnostic sample acquisition was also evaluated, and only those samples collected by cystocentesis or catheter assistance were utilized for the study. Following medical record review and isolate exclusion, patients were categorized as exposed and unexposed to antibiotics based on recorded treatment history.

### Antibiotic resistance phenotype characterization.

The MICs to a panel of antibiotic drugs important to human and veterinary medicine were determined using the Sensititre Vizion (CMV4AGNF and COMPGN1F panels; Thermo Fisher Scientific, Oakwood Village, OH) broth microdilution system following Clinical and Laboratory Standards Institute (CLSI) guidelines (40). Isolates were classified as susceptible or reduced susceptible, including those with an intermediate or resistant classification, using CLSI breakpoints (40). E. coli strains ATCC 25922 and ATCC 35218 were used as quality controls. Multidrug reduced susceptibility was defined as reduced susceptibility to at least one antibiotic from three or more drug categories (41).

### Whole-genome sequencing and molecular pathogenomics.

All isolates underwent short-read sequencing using the Illumina MiSeq platform. Sequences were quality assessed and adapter sequences were trimmed using FastQC and TrimGalore, respectively (42, 43). Only whole-genome sequences with a minimum sequencing depth of 50× were considered for assembly. The resulting sequences were assembled using Unicycler (44). Assembly quality was assessed using QUAST, and only those assemblies with >99% reference genome completeness, <0.5% sequence contamination, contig *N*_50_ of >99.5%, and percent protein-encoding-feature coverage of >95% were included in this study (45). High-quality assemblies were annotated using Prokka and the PGAP pipeline (46, 47). Assembled sequences were assessed for acquired antibiotic resistance genes using the ResFinder 4.1 (https://cge.food.dtu.dk/services/ResFinder/) online database with default parameters from the Center for Genomic Epidemiology (48). Annotated contigs were compared to a library of individual E. coli virulence genes using the Ecol_VF library within the ABRICATE package (49). Virulence factors were considered present if there was at least 80% coverage and 80% identity to the reference sequence or protein.

### Population structure characterization.

Isolates were further characterized into sequence types and sequence type complexes using the Achtman multilocus sequence typing (MLST) criteria via the PubMLST online database (https://pubmlst.org) (50). Isolate phylogroup was assigned using the *in silico* Clermont phylotyping tool (http://clermontyping.iame-research.center) (51). A *de novo* core-genome single-nucleotide-polymorphism analysis and maximum-likelihood phylogenetic tree were constructed using CSIPhylogeny version 1.4 (https://cge.food.dtu.dk/services/CSIPhylogeny/) with default settings (52). E. coli K-12 strain MG1655 (GenBank accession number LR881938) was used as the reference strain. Phylogenetic trees were visualized using the Interactive Tree of Life (iTOL) interface (https://itol.embl.de) (53).

### Data analysis.

Differences in clinical and demographic information between the exposed and unexposed groups and UPEC phylogroups—dichotomized as phylogroup B2 and non-B2—were compared using generalized estimating equations to construct univariate logistic regression models while controlling for repeat submissions from the nine dogs that submitted repeat samples. In addition to this model, we used generalized estimating equations to train regression models to estimate the associations between clinical and demographic variables, antibiotic exposure status, and UPEC phylogroup and the UPEC virulome, resistome, and antibiotic susceptibility while controlling for dogs that submitted repeat samples. The type of regression used was dependent on the distribution of the outcome variable: antibiotic susceptibility models used logistic regression, resistome models used a negative binomial regression, and virulome used a linear regression. If more than one predictor was significant (*P* < 0.05) in the univariate analysis, we employed a forward-selection model building approach to construct multivariate models to calculate adjusted associations and their *P* values (54). Final models were tested for parsimony using a likelihood-ratio test.

The quantities and proportional differences of virulence and antibiotic resistance genes were associated with antibiotic exposure and phylogroup using the Wilcoxon rank sum test and Fisher’s exact test, respectively. The Benjamini-Hochberg method was used to adjust *P* values for multiple comparisons. Those values with an adjusted *P* value of <0.1 were considered statistically significant and included for data visualization. Principle coordinate analysis was constructed using Base R to generate Bray-Curtis dissimilarity distance matrices. The dissimilarity matrix was input into Vegan in R, where the principal coordinate plot was constructed and visualized using the top two eigenvectors (55). All statistical analysis was conducted using STATA version 15 (StataCorp LLC, College Station, TX, USA), and data were visualized using the R package ggplot2 (https://ggplot2.tidyverse.org) (56).

### Data availability.

Genome sequences were submitted to GenBank and are available under BioProject accession number PRJNA773999.
